# Gliflozins Have an Anti-Inflammatory Effect on Renal Proximal Tubular Epithelial Cells in a Diabetic and Inflammatory Microenvironment In Vitro

**DOI:** 10.3390/ijms24031811

**Published:** 2023-01-17

**Authors:** Benjamin Koch, Dominik C. Fuhrmann, Ralf Schubert, Helmut Geiger, Thimoteus Speer, Patrick C. Baer

**Affiliations:** 1 Department of Internal Medicine 4, Nephrology, University Hospital, Goethe-University, 60596 Frankfurt, Germany; 2 Institute of Biochemistry I, Faculty of Medicine, Goethe-University, 60590 Frankfurt, Germany; 3 Division of Allergology, Pneumology and Cystic Fibrosis, Department for Children and Adolescents, University Hospital, Goethe-University, 60596 Frankfurt, Germany

**Keywords:** renal tubular cells, epithelial cells, proximal tubule, cytokines, inflammation, empagliflozin, dapagliflozin, kidney, ICAM-1

## Abstract

Inflammation is intimately involved in the pathogenesis of diabetic kidney disease. Inhibition of SGLT-2 by a specific class of drugs, gliflozins, has been shown to reduce inflammation and attenuate the progression of diabetic nephropathy, in addition to its main effect of inhibiting renal glucose reabsorption. We used highly purified human renal proximal tubular epithelial cells (PTCs) as an in vitro model to study the cellular response to a diabetic (high glucose) and inflammatory (cytokines) microenvironment and the effect of gliflozins. In this context, we investigated the influence of SGLT-2 inhibition by empa- and dapagliflozin (500 nM) on the expression of pro-inflammatory factors (IL-1β, IL-6, TNF-α, MCP-1, and ICAM-1). The results clearly indicate an anti-inflammatory effect of both gliflozins. Although induced expression of the four cytokines was only slightly attenuated, there was a clear effect on the expression of the adhesion molecule ICAM-1, a master regulator of cellular responses in inflammation and injury resolution. The induced expression of ICAM-1 mRNA was significantly reduced by approximately 13.5% by empagliflozin and also showed an inhibitory trend with dapagliflozin. However, induced ICAM-1 protein expression was significantly inhibited from 24.71 ± 1.0 ng/mL to 18.81 ± 3.9 (empagliflozin) and 19.62 ± 2.1 ng/mL (dapagliflozin). In conclusion, an additional anti-inflammatory effect of empa- and dapagliflozin in therapeutically observed concentrations was demonstrated in primary human PTCs in vitro.

## 1. Introduction

Gliflozins are a class of antidiabetic drugs based on the selective inhibition of the sodium-glucose co-transporter 2 (SGLT-2) that increase urinary glucose excretion [1]. Inhibition of SGLT-2 blocks the reabsorption of glomerular-filtered glucose (and sodium) in the renal proximal tubular segment, a mechanism used to reduce hyperglycemia in patients with type 2 diabetes mellitus [2]. Gliflozins have also been shown to have favorable effects on body weight, blood pressure, lipid profile, arterial stiffness, and endothelial function, and can reduce glycated hemoglobin [3]. In recent years, the renoprotective function of gliflozins has been reported in diabetic nephropathy, which occurs in approximately 40% of patients with diabetes and is a common cause of chronic kidney disease worldwide [4]. Among the various kidney diseases, diabetic nephropathy is one of the most common causes of end-stage renal disease, which is responsible for the morbidity and high mortality rate in patients with diabetes [5]. The protective effect of SGLT-2 inhibition on reducing acute and chronic cardiovascular events is also well known [6]. A number of large clinical trials have been conducted to evaluate the safety and efficacy of gliflozins in patients with diabetes and established vascular disease, multiple cardiovascular risk factors, or renal failure, and in patients with established heart failure and reduced ejection fraction [6]. In addition to the structural differences between the different clinically approved gliflozins, the agents have different half-lives and selectivity for the transporter [6]. 

Inflammation is closely involved in the pathogenesis of diabetic kidney disease. The inhibition of SGLT-2 has been described to reduce inflammation and attenuate the progression of diabetic nephropathy [7]. It is well known that a diabetic microenvironment triggers inflammation and increases oxidative stress in tissues and organs affected by diabetes. The mechanisms leading to the excessive stimulation of inflammation and oxidative stress were reviewed recently in a series of papers [8,9]. In addition, empagliflozin has been shown to exert anti-inflammatory and anti-fibrotic effects on diabetic nephropathy in vivo, in part by suppressing the formation of advanced glycation end products and oxidative stress generation in the kidneys [10]. Other mechanisms of SGLT-2 inhibitors that may prove beneficial include a reduction in adipose tissue-mediated inflammation and the production of pro-inflammatory cytokines, a shift to ketone bodies as a metabolic substrate for the heart and kidneys, a reduction in oxidative stress, a reduction in serum uric acid levels, a reduction in glomerular hyperfiltration and albuminuria, and a suppression of advanced glycation end-product signaling [11].

In our current project, highly purified human renal proximal tubular epithelial cells (PTCs) were used as an in vitro model to study the cellular response to high glucose in combination with an inflammatory microenvironment and the effect of gliflozins during this response. To the best of our knowledge, this is the first study using primary highly differentiated PTCs to investigate the possible effects of two clinically used gliflozins: empa- and dapagliflozin. Whether SGLT-2 inhibitors can directly protect the renal tubular epithelium from damage and inflammation caused by high glucose is currently not fully investigated. Because the proximal tubular epithelium is the direct site of action of SGLT-2 inhibitors, a simulation of several environmental variables, high glucose, and inflammation, was used to examine the direct effect on this cell type in vitro and possibly simulate conditions in the human nephron during diabetic nephropathy.

## 2. Results

### 2.1. Measurement of Cytotoxicity and Oxidative Stress

**Figure 1:**
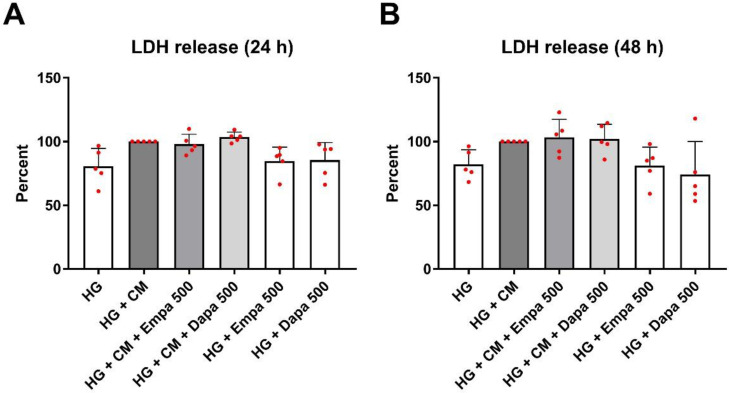
Cytotoxic effects under diabetic and inflammatory culture conditions (lactate dehydrogenase (LDH) assay). The measurement of LDH activity released from the cytosol of damaged cells into the supernatant was used for the quantification of cell death and lysis. The PTCs cultured in 96-well plates were incubated for 24 (**A**) or 48 h (**B**) in media containing empa- or dapagliflozin under a diabetic (high glucose) or diabetic in combination with an inflammatory (cytokine mix, CM) environment. No significant effects of the different treatments on cytotoxicity were detected (analysis of variance (ANOVA) with Dunnett’s multiple comparison test). Absorbance was measured in a microplate reader (490 vs. 650 nm, arbitrary units, calculated as a percent in relation to the control (HG + CM), scatter plots with bar, mean ± SD, *n* = 5).

To test for cytotoxic effects that are caused by the stimulations, a quantification of lactate dehydrogenase (LDH) in the supernatant of PTCs was performed. We detected no cytotoxic effects after 24 or 48 h of incubation in the different media formulations (Figure 1). This was to be expected due to the short stimulation periods (24 (Figure 1A) and 48 h (Figure 1B)). Nevertheless, a small trend between HG and HG + CM could be seen, which is consistent with our previous results after 6 days of stimulation [12]. Here, a significant cytotoxic effect of the cytokine mix (CM) was detected.

In addition, the formation of intracellular oxidative stress was tested using fluorescence measurements of intracellular 2′,7′-dichlorofluorescein (DCF). Unloaded cells were used as negative controls and compared with cells after DCF loading (loading controls). In addition, at the end of each experiment, we added H_2_O_2_ (250 µM) to each well, resulting in a maximum signal (positive control).

**Figure 2:**
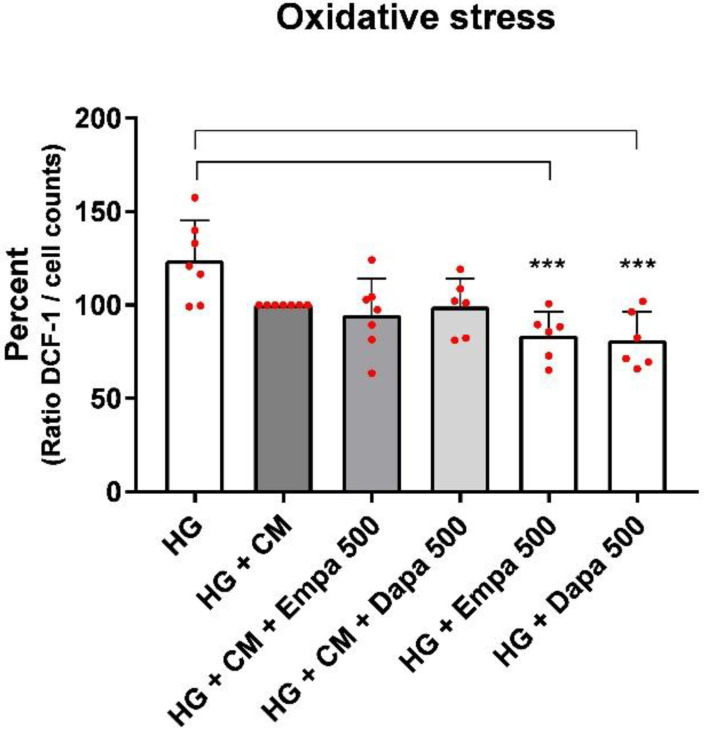
Formation of oxidative stress under diabetic and inflammatory culture conditions (DCF assay). The PTCs cultured in 96-well plates were incubated for 48 h in media containing empa- or dapagliflozin (500 nm) under a diabetic (high glucose) or diabetic in combination with an inflammatory (cytokine mix, CM) environment. Then, 2′,7′-dichlorodihydrofluorescein diacetate (20 µM in Hank’s buffered saline solution) was added for 30 min at 37 °C. Fluorescence of intracellular DCF was measured using a fluorescence reader with excitation and emission wavelengths of 485 and 538 nm, normalized to the cell counts, and the ratio was calculated as a percent (scatter plots with bar, mean ± SD, *n* = 6–7, *** *p <* 0.001 vs. HG).

Incubation of PTCs in HG with gliflozins for 48 h reduced the oxidative stress levels compared to HG alone in a significant manner (Figure 2). The results are in line with our previous results after 6 d of incubation [12]. Nevertheless, this effect of the gliflozins was only detectable in the high-glucose medium and not in the high-glucose medium with the addition of cytokines. Surprisingly, the addition of the cytokine mix (HG + CM) induced a low level but not a significant decrease in oxidative stress levels compared to the diabetic medium (Figure 2). It was also statistically tested whether the HG + CM versus the HG + CM + gliflozin treatment was significantly changed. No significance was demonstrated here.

### 2.2. Effect on the Expression of Pro-Inflammatory Cytokines

**Figure 3:**
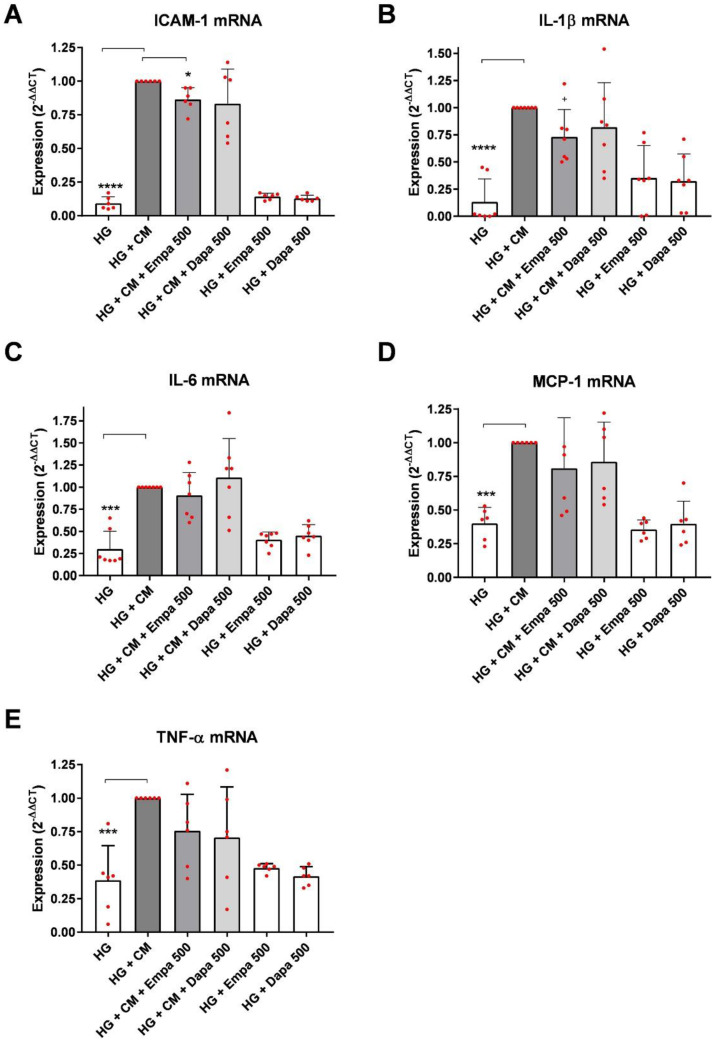
Effect of empagliflozin and dapagliflozin on mRNA expression of selected factors under diabetic and inflammatory culture conditions, (**A**) ICAM-1, (**B**) IL-1β, (**C**) IL-6, (**D**) MCP-1, (**E**) TNF-α. Cells were grown in small culture flasks until confluence, serum depleted for 2 h, and cultured as indicated for 24 h. The expression levels in each experiment were normalized to a housekeeping gene (β-actin) and expressed relative to the control using the ∆∆CT method. A mixture of pro-inflammatory cytokines (CM) significantly increased the mRNA expression of all five readouts after 24 h incubation. Scatter plots with bar, mean ± SD, **** *p* < 0.0001 (**A**,**B**), *** *p* < 0.001 (**C**–**E**), * = 0.0217 (**A**), + = 0.0519 (**B**), *n* = 6–7.

In the next step, we investigated the influence of SGLT-2 inhibition by gliflozins under the diabetic and inflammatory microenvironment on mRNA expression of pro-inflammatory factors. We checked the mRNA levels of the pro-inflammatory cytokines interleukin (IL)-1β, IL-6, and TNF-α; the monocyte chemotactic protein-1 (MCP-1); and the intercellular adhesion molecule-1 (ICAM-1) after 24 h of incubation. As shown in Figure 3, the expressions of all five readouts could be significantly induced by adding a cytokine mix and thus creating an inflammatory microenvironment. Additional incubation with either empagliflozin or dapagliflozin did not significantly affect the mRNA expression of the three cytokines (Figure 3B,C,E) and the chemokine (Figure 3D). However, there was a clear trend toward an inhibitory effect. In the diabetic and inflammatory microenvironment, both gliflozins decreased the expression of the cytokines. However, only the induced expression of the cell adhesion molecule ICAM-1 was significantly reduced by empagliflozin (HG + CM + empa: 0.865 ± 0.08 (=13.5 %), *p* = 0.0217; Figure 3A). Dapagliflozin also tended to reduce this expression but not significantly (HG + CM + Dapa: 0.833 ± 0.25, Figure 3A). The addition of the gliflozins in the diabetic microenvironment induced by a high-glucose medium (and without additional inflammation) did not influence the expression of the five readouts compared to the diabetic medium alone (HG) (Figure 3A–E).

**Figure 4:**
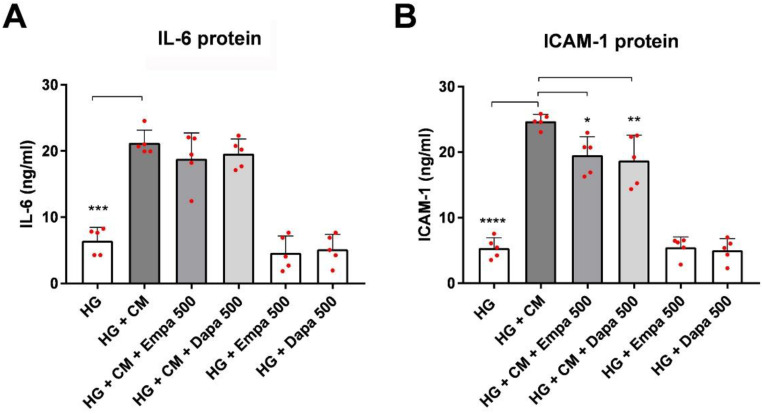
Effect of empagliflozin and dapagliflozin on the release of (**A**) IL-6 and (**B**) ICAM-1 protein. Cells were grown in a 24-well plate until confluence, serum depleted for 2 h, and cultured in the media indicated. Supernatants were then harvested and quantified using commercially available immunoassays (optical density 450/620 nm, scatter plots with mean ± SD). (**A**) *** *p* < 0.01 versus HG, *n* = 5, (**B**) * = 0.019, ** = 0.005, **** *p* < 0.001, *n* = 5.

Then, we checked the release of IL-6 and ICAM-1 protein in the supernatant of the PTCs (Figure 4A,B). At the protein level, IL-6 and ICAM-1 were constitutively expressed by PTCs cultured in HG (IL-6: 6.46 ± 1.9 ng/mL; ICAM-1: 5.38 ± 1.5 ng/mL; mean ± SD, *n* = 5). Incubation in the presence of the cytokine mix (CM) significantly increased the release of both proteins (IL-6: 21.23 ± 1.9 ng/mL; ICAM-1: 24.71 ± 1.0 ng/mL; mean ± SD, *n* =5). No significant inhibitory effect on IL-6 release was detected after co-incubation of CM with either empa- or dapagliflozin compared to CM alone (HG + CM + empa: 18.81 ± 3.9 ng/mL; HG + CM + Dapa 19.62 ± 2.1 ng/mL; mean ± SD, *n* =5).

## 3. Discussion

The use of SGLT-2 inhibitors has been increasingly extended to prevent the progression of chronic kidney disease and heart failure, even in patients without type 2 diabetes. Most mechanistic studies of SGLT-2 inhibition have focused on a range of hemodynamic effects, precisely because of the benefits in terms of heart failure and renal end points. The mechanisms explaining the benefits of the various gliflozins are still the subject of investigation. Nevertheless, the underlying molecular mechanisms are still not completely understood. Considering the described close interplay between inflammation and metabolic deregulation and the beneficial effects of SGLT-2 inhibition observed in diseases with metabolic and inflammatory disorders (type 2 diabetes, heart failure, diabetic nephropathy, diabetic cardiomyopathy), it is natural to investigate its role as a therapeutic agent with anti-inflammatory effects [13]. The inhibition of SGLT-2 has also been associated with reduced inflammation in diabetes models and decreased inflammasome activation in the kidneys and heart [14,15,16]. Ishibashi and coworkers have shown that SGLT-2-mediated glucose entry into tubular cells can stimulate oxidative stress, monocyte chemoattractant protein-1 expression, and apoptotic death and that tofogliflozin blocked these effects in vitro [5]. Exposure of a cultured renal cell line to a high-glucose concentration also resulted in a significant increase in reactive oxygen species. This increase could be prevented by adding dapagliflozin [17], empagliflozin [18], or canagliflozin [19]. Other studies have shown that high glucose increased the nuclear translocation of nuclear factor-κB p65 and the expression of cytoplasmic nucleotide-binding oligomerization domain-like receptor 3 (NLRP3), interleukin-1β, interleukin-6, and tumor necrosis factor-α (TNF-α) [20]. Dapagliflozin reduced the extent of these HG-induced inflammatory alterations in a cell line in vitro [20]. Das and coworkers demonstrated that empagliflozin suppresses HG-induced intracellular signal transduction and inflammatory cytokine expression in a cell line in vitro [18]. Several studies also investigated the anti-inflammatory effects of gliflozins in vivo (reviewed in [6,9]). Treatments in diabetic models clearly showed improvements in histopathological examinations and inflammatory and apoptotic markers.

Furthermore, gliflozins have been shown to improve cortical renal hypoxia by ameliorating excessive oxygen and ATP consumption in a diabetic mice model [21]. The SGLT-2 inhibitors’ pleiotropic effects have also been demonstrated by the suppression of high-glucose-induced angiotensinogen augmentation in proximal tubular [19] and endothelial cells [22]. In addition, dapagliflozin has been revealed to restore autophagic flux and suppress NF-kB’s subunit p65 in proximal tubular epithelial cells exposed to a high-glucose medium [20]. Moreover, empagliflozin has been indicated to reduce oxidative stress-induced damage (by activation of the Akt/GSK-3 signaling pathway) and target inflammation and fibrosis by the downregulation of TGF-ß1 in a model of diabetic nephropathy [23]. Besides the reduction in oxidative stress and inflammation, Malínská and coworkers found a reduced lipid deposition in the kidneys of spontaneously hypertensive rats expressing human C-reactive protein [24]. In the present study, we focus on the effects of two currently available gliflozins on renal proximal tubular epithelial cells. In detail, we examined the anti-inflammatory effects of empa- and dapagliflozin on primary and highly differentiated PTCs under a diabetic and inflammatory microenvironment in vitro. Importantly, diabetes itself is a systemic pro-inflammatory condition, involving not only the kidneys, but also the cardiovascular system and bones [25,26]. High levels of circulating glucose and inflammatory cytokines are the main causes of tissue damage and reactive oxygen species in diabetes mellitus by inducing cell injury in the kidneys [27]. In the case of circulating cytokines in diabetes, for example, γ-interferon expression showed an increase of 290 pg/mL [28]. Compared to the pro-inflammatory in vitro system used in this study, this concentration is over tenfold higher. We used γ-interferon at 200 U/mL, which is equivalent to 20 pg/mL. However, we employed a cytokine mix with two other cytokines in the stimulations. Our study clearly demonstrates an anti-inflammatory effect of empa- and dapagliflozin. Induced ICAM-1 mRNA and protein expression levels were shown to be significantly inhibited by both gliflozines. However, this induced mRNA expression was significantly reduced by the gliflozines for only one of the five pro-inflammatory molecules examined. This is certainly due to fluctuating values and thus to the high standard deviation. Nevertheless, there was also a clear tendency toward inhibited expression for the four other readouts. The results agree well with previous studies showing that SGLT-2 inhibition can achieve its nephroprotective effect by attenuating the chronic inflammatory process. In this context, several studies have shown that inflammation and oxidative stress promote renal tubular epithelial and interstitial fibrosis, resulting in irreversible renal damage, and, consequently, end-stage nephropathy [18,29,30]. 

ICAM-1 is a cell surface glycoprotein and an adhesion receptor, which is responsible for the recruitment of leukocytes to sites of inflammation [31]. ICAM-1 is expressed at a low basal level in immune, endothelial, and epithelial cells, but is upregulated in response to inflammatory stimulation and is described as a master regulator of cellular responses in inflammation and injury resolution [31]. Several studies investigated the anti-inflammatory effects of SGLT-2 inhibitors using different experimental approaches. In vitro studies used immortalized tubular epithelial cells (e.g., HK-2 cell line) [32,33,34,35], endothelial cells [36], macrophages [13], or other cells [37] to investigate the anti-inflammatory (and anti-oxidative) effects of different gliflozins. Yao and coworkers investigated the effects of dapagliflozin on immortalized human kidney (HK-2) cells in a diabetic environment and described that ICAM-1 mRNA and protein expression levels increased in high-glucose conditions and could be reduced by dapagliflozin [32]. Interestingly, others described that empagliflozin did not influence TNF-a-induced ICAM-1 expression in human endothelial cells in vitro [38]. Pirkbauer et al. demonstrated at the transcriptome level that empagliflozin attenuates the expression of several inflammatory response genes in IL-1β-induced renal epithelial cell lines in a normoglycemic environment [39]. Previous data from this group provided evidence for the anti-inflammatory effects of empagliflozin in renal epithelial cell lines by demonstrating inhibition of IL-1β-induced MCP-1 and endothelin-1 expression at the mRNA and protein levels [40]. Incubation with clinically relevant concentrations of canagliflozin, but not dapagliflozin or empagliflozin, was shown to inhibit IL-1β-stimulated adhesion of monocytic cells to endothelial cells in vitro and the release of IL-6 and MCP-1 [41]. Empagliflozin has also been shown to inhibit COX-2 and iNOS gene expression and prostaglandin E2 release in LPS-stimulated macrophages [42]. Furthermore, the empagliflozin dose dependently inhibited cytokine-induced CXCL10 secretion in human cardiomyocytes but not in skeletal muscle cells [43]. The inhibition in cardiomyocytes was dose dependent (IC_50_ = 80 nM) and associated with a decrease in cytokine-induced phosphorylation of the transcription factor Stat-1. Because of the different effects on cardiomyocytes and skeletal muscle cells, Giannattasio et al. suggested empagliflozin-selective cell targeting in their model [43], although this cell-type targeting should be verified in further models. However, although gliflozins’ SGLT-2-based actions are self-evident by SGLT-2′s tissue distribution [44], the observed pleiotropic impacts imply numerous (beneficial) off-target effects [45]. Others demonstrated that empagliflozin can ameliorate the LPS-induced mRNA expression of IL-6, TNF-α, and IL-1β in primary microglial cells [37]. This study also demonstrated a reduced protein release of IL-6, TNF-α, and IL-10 after empagliflozin incubation. Kolijn and coworkers described a significantly reduced microvascular inflammation after empagliflozin treatment in vivo [46]. Empagliflozin has been shown to significantly suppress increased levels of ICAM-1, VCAM-1, TNF-α, and IL-6 in the human and rat myocardium. It also attenuated pathological oxidative stress in cardiomyocytes in addition to improved endothelial vasorelaxation. In contrast, in another study dapa-, empa-, and canagliflozin did not prevent an increase in the mRNA expression of IL-6, IL-8, and IL-1β (induced by LPS in monocytes or by hyperglycemia in endothelial cells), suggesting that these drugs do not have intrinsic anti-inflammatory properties in these situations [47].

It should also be noted that the gliflozin concentrations used in the various in vitro studies varied widely. Some studies used so-called supra-pharmacological concentrations up to 100 µM [32,38,42,48]. We incubated the cells with empa- and dapagliflozin in therapeutically possible concentrations (500 nM). The concentrations of both gliflozins matched the published peak therapeutic serum concentration (C_max_ ~ 500 nM) [39,40]. At this concentration, no direct cytotoxicity/cell injury in cultured primary renal proximal tubular cells was detected, either in long-term incubation in a diabetic medium [14] or short-term-stimulation in a diabetic and pro-inflammatory microenvironment (current study, shown by LDH release).

In conclusion, our current study provides further insights into the anti-inflammatory effects of dapa- and empagliflozin. Our results demonstrate, in the context of numerous recent reports, additional positive gliflozin effects on the renal epithelium, thus complementing their main effect, that is, the reduction in glucose (and sodium) reabsorption via SGLT-2 inhibition.

## 4. Materials and Methods

### 4.1. Isolation, Culture, and Characterization of Human Renal Proximal Tubular Epithelial Cells

Human renal proximal tubular epithelial cells (PTCs) were isolated as described previously [49]. In brief, PTCs were separated after tumor nephrectomies from renal tissue not involved in cell carcinoma. Kidney tissue was minced by crossed blades, then digested with collagenase/dispase. The tissue was then filtered through a mesh (106 µm), redigested with collagenase IV, DNase, and MgCl_2,_ and centrifuged using a Percoll density gradient. Finally, highly purified PTCs were separated immunomagnetically using a mAb against aminopeptidase M (CD13) and the Mini-MACS system (Miltenyi, Germany). Primary isolates were strongly positive for aminopeptidase M and ultrastructural analysis revealed well-preserved brush border microvilli, a well-developed endocytosis apparatus, and numerous mitochondria [49,50]. PTCs were cultured in a standard culture medium (Medium 199 (M4530, Sigma, Taufkirchen, Germany) with a physiological glucose concentration (100 mg/dL) and with 10% fetal bovine serum (FBS; Biochrom, Berlin, Germany)). The medium was renewed every three to four days. Trypsinization was used to passage confluent cells, and passages 2 and 5 were used in this study. Cultured PTCs were characterized, as described previously [49,50]. In addition, the SGLT-2 mRNA and protein expressions of cultured PTCs were shown by PCR analysis and immunofluorescence staining [12]. Glucose uptake in PTCs was shown by 2-Deoxy-2-[(7-nitro-2,1,3-benzoxadiazol-7-yl)amino]-D-glucose (NBDG-2), a fluorescent glucose analogon used in fluorescence measurements [12]. 

### 4.2. Stimulations 

PTCs were proliferated to confluence in a standard cell culture medium (as described in Section 4.1). PTCs were then washed, placed in serum-free Medium 199 for 2 h, and stimulated as indicated in the respective assay. Stimulations were performed in high-glucose Medium 199 (HG, glucose content 450 mg/dL [25 mM]) with 10% FBS, which induced a diabetic milieu in the cultures. 

For protein measurements in the supernatants (EIA, Section 4.6), stimulations were performed in HG without serum. For stimulations, empagliflozin (Adipogen, San Diego, CA, USA; No. AG-CR1-3619) and dapagliflozin (Cayman Chemical, Ann Arbor, MI, USA; No. 11574) were dissolved in dimethylsulfoxide (stock solution 1 mM) and diluted in the medium to 500 nM. This concentration of both gliflozins (500 nM) matched the published maximal therapeutic concentration observed (C_max_) [51,52]. An inflammatory microenvironment was induced by culturing PTCs in HG with a mixture of cytokines (CM) containing γ-interferon (200 U/mL), interleukin-1β (25 U/mL), and TNF-α (10 ng/mL). Stimulations were performed for 24 or 48 h, as indicated.

### 4.3. Cytotoxicity Assay 

Measurement of LDH (Sigma Aldrich, Darmstadt, Germany, No. 11644793001) released from the cytosol of damaged cells into the supernatant was used to quantify cell death and cell lysis to determine the cytotoxic effects. For this purpose, cell culture supernatants were harvested after 24 and 48 h incubation. As described by the manufacturer, absorbance was measured in a microplate reader at 490 vs. 650 nm. Data were calculated as a percentage relative to stimulation in the inflammatory environment (HG + CM = 100%).

### 4.4. Measurement of Oxidative Stress 

To detect reactive oxygen species in PTCs, the cell-permeant 2′,7′-dichlorodihydrofluorescein diacetate (H_2_DCFDA) was used. Nonfluorescent H2DCFDA is converted to highly fluorescent DCF in cells by cleavage of the acetate groups by intracellular esterases and oxidation. For the experiments, confluent PTCs were cultured for 48 h in HG, CM, or with gliflozines (500 nM). The cells were then washed with pre-warmed HBSS and H_2_DCFDA (20 µM in HBSS) was added for 30 min at 37 °C (in quintuplicate for each biological replicate). PTCs processed in the buffer without DCF were used as background controls. Finally, cells were washed with HBSS, and fluorescence was measured immediately using a fluorescence reader (BMG Fluostar, Ortenberg, Germany), with excitation and emission wavelengths of 485 and 538 nm, respectively. Data were expressed as the ratio of arbitrary fluorescence units normalized against cell counts.

### 4.5. PCR 

RNA extraction was performed using single-step isolation following standard protocol. After RNA extraction, cDNAs were transcribed for 30 min at 37 °C using 1 µg RNA, 50 µM random hexamers, 1 mM deoxynucleotide triphosphate mix, 50 units reverse transcriptase (Fermentas, St. Leon-Rot, Germany) in 10× PCR buffer, 1 mM β-mercaptoethanol, and 5 mM MgCl_2_. A Hot FIREPol EvaGreen Mix Plus (Solis Biodyne, Tartu, Estonia) was used for the master mix; the primer mix and RNAse-free water were added. Quantitative PCR was carried out under the following conditions: twelve minutes at 95 °C for enzyme activation, 15 s at 95 °C for denaturation, 20 s at 63 °C for annealing, and 30 s at 72 °C for elongation (40 cycles). In addition, a melting curve analysis was carried out, as well as agarose gel electrophoresis in selected experiments, to check the products. For the quantification of the PCR fragments, we used the ABI Prism^®^ 7900HT Fast Real-Time PCR System with a Sequence Detection System SDS 2.4.1 (Thermo Fisher Scientific). Relative quantification was then carried out with the ∆∆CT method [53] using β-actin as a housekeeper, and the level of target gene expression was calculated using 2^−∆∆*C*t^. In selected experiments, PCR products were separated by agarose electrophoresis (2%) and observed under ultraviolet illumination. Primer pairs were synthesized using Invitrogen (Karlsruhe, Germany) and are listed in Table 1. 

### 4.6. Immunoassays 

Cell culture supernatants were harvested after stimulation for 48 h (described in Section 4.2), centrifuged at 300× *g* for 5 min, and used for the quantification of IL-6 and ICAM-1, or stored at −20 °C for later measurement. Both proteins were quantified using commercially available EIA kits (IL-6: Immunotools, Friesoythe, Germany; No. 31670069; ICAM-1 R&DSystems, Wiesbaden, Germany; No. DY720-05), as described by the manufacturer. In brief, the wells were coated with the capture antibody overnight at room temperature. Then, nonspecific binding sites were blocked with a blocking buffer for 1 h and washed with PBS/0.05 % Tween. The standard (IL-6 assay: 8–500 pg/mL, ICAM-1: 31.1-2000 pg/mL) or the samples were added for 2 h at room temperature. All samples were diluted in assay buffer (IL-6 1:25; ICAM-1 1:10) and run in duplicate. After incubation, the plates were washed and the biotinylated detection antibody was added for 2 h at room temperature, washed again, and incubated with horseradish-peroxidase-streptavidin for an additional 30 min. After washing, the substrate Tetramethylbenzidine was added for 5–20 min and the reaction was stopped and measured (450 vs. 620 nm). The data are calculated as ng/mL in the supernatant.

### 4.7. Statistical Analysis 

Analysis of variance (ANOVA) with Dunnett’s multiple comparison test was used for statistical analysis. Gaussian distribution of the data was tested with the Shapiro–Wilk test. The data are expressed as mean ± SD. *p* values < 0.05 were considered significant. The statistical analysis of the measured data, as well as their graphic representation, was performed using Prism software, version 7.04 (GraphPad, Boston, MA, USA). 

## Figures and Tables

**Table 1:** Primer used for PCR analyses.

Gene	Primer Forward	Primer Reverse	Product Length (bp)	NCBI Reference Sequence
ICAM-1	CAACCTCAGCCTCGCTATGG	CGGGGCAGGATGACTTTTGA	135	NM_003041.3
IL-6	AAA GAT GGC TGA AAA AGA TGG ATG C	ACA GCT CTG GCT TGT TCC TCA CTA C	150	NM_000600.4
IL-1β	AGCTGATGGCCCTAAACAGA	AGATTCGTAGCTGGATGCCG	83	NM_000576.3
MCP-1	CCCCAGTCACCTGCTGTTAT	AGATCTCCTTGGCCACAATG	135	NM_002982.4
TNF-α	CGGGACGTGGAGCTGGCCGAGGAG	CACCAGCTGGTTATCTCTCAGCTC	354	NM_000594.4
β-Actin	ACT GGA ACG GTG AAG GGT GAC	AGA GAA GTG GGG TGG CTT TT	169	NM_001101

## Data Availability

Not applicable.
